# Anatomical layer analysis for safe thread insertion using micro-CT and ultrasonography: enhancing precision in facial thread-lifting procedures

**DOI:** 10.3389/fmed.2026.1581406

**Published:** 2026-01-28

**Authors:** Kyu-Ho Yi, Hyung-Jin Lee, Ji-Hyun Lee

**Affiliations:** 1 Division in Anatomy and Developmental Biology, Department of Oral Biology, Human Identification Research Institute, BK21 FOUR Project, Yonsei University College of Dentistry, Seoul, Republic of Korea; 2 Department of Anatomy, College of Medicine, Chungbuk National University, Cheongju, Republic of Korea; 3 Department of Anatomy and Acupoint, College of Korean Medicine, Gachon University, Seongnam, Republic of Korea

**Keywords:** micro-CT, post-procedure identification, SMAS layer, thread lifting, ultrasonography

## Abstract

**Background:**

Thread lifting is considered a relatively noninvasive and effective treatment for facial rejuvenation compared to traditional facelift surgery. However, no consensus exists on anatomically favorable insertion layer for threads into the lateral facial area. This study aimed to examine the anatomy of the lateral face, particularly the superficial musculoaponeurotic system, and facial nerve course, and evaluate the safety of thread insertion into this region.

**Materials and methods:**

Eight fresh cadavers were used. Thread insertion was performed in the lateral face with a single entry point at the temple area. Three threads were inserted: one terminated 1 cm lateral to the mouth corner, and the other terminated 1 cm lateral to the first termination point on either side. The safety of thread insertion was assessed using two advanced imaging modalities—micro-computed tomography with the contrast-enhanced agent phosphotungstic acid and ultrasonography.

**Results:**

The threads were successfully inserted into the correct layer, superficial to the superficial musculoaponeurotic system, without damaging critical anatomical structures such as vessels, nerves, or muscles in the lateral face area.

**Conclusion:**

This study demonstrated that thread insertion into the superficial layer of the superficial musculoaponeurotic system is anatomically favorable approach for lifting lateral facial tissues. This layer offers the potential for achieving feasible results while minimizing iatrogenic complications in thread-lifting procedures.

## Introduction

Facial thread lifting is a widely utilized esthetic procedure to rejuvenate the face by lifting sagging tissue and redefining contours. It has gained popularity due to its minimally invasive nature and ability to lift and tighten facial tissues without requiring surgery. However, the procedure’s safety and efficacy largely depend on accurately placing the threads in the correct anatomical layer. Inappropriate thread placement may lead to complications such as thread migration, dimpling, asymmetry, or even damage to critical structures, including the superficial temporal artery, transverse facial artery and its perforators, facial nerve branches, and parotid duct (1–3).

The face comprises multiple layers, including the skin, subcutaneous tissue, facial muscles, the superficial musculoaponeurotic system (SMAS), and deeper muscle or fatty layers. Each layer presents different mechanical properties and risks when penetrated by threads. For feasible results, threads are generally recommended to target within or just above the SMAS layer, where tissue resistance and anchoring capacity are sufficient for effective lifting while minimizing the risk of complications (4, 5). However, threads placed too superficially in the subcutaneous layer may bulge and lead to esthetically undesirable outcomes. Conversely, overly deep placement beneath the SMAS layer may risk damage to critical structures such as the facial nerve, transverse facial artery, or parotid duct. Variability in facial anatomy across age, sex, ethnicity, and individuals highlights the need to identify a safe anatomical layer for thread insertion, as no consensus currently exists regarding the optimal plane for placement. Clinical practitioners often rely on individual techniques and experiences, which may result in inconsistent outcomes or complications.

Modern imaging techniques, such as micro-computed tomography (micro-CT), provide valuable tools for analyzing thread placement. Micro-CT allows for high-resolution visualization of anatomical structures and thread positioning and its spatial relationship with surrounding anatomical structures post-procedure. These methods offer a comprehensive approach to understanding and refining thread-lifting techniques (6–8).

This study aims to determine a safe anatomical layer for thread placement by analyzing insertion planes, assessing safety considerations, and examining post-placement thread positioning in cadaveric models. Furthermore, we seek to establish evidence-based recommendations for safer and more effective thread-lifting procedures, ultimately contributing to improved clinical outcomes and patient satisfaction.

## Materials and methods

### Specimen procurement

A total of eight fresh-frozen cadavers (five males and three females; mean age, 72.1 years) were utilized. All cadavers were allocated for ultrasonography and micro-CT evaluation of thread insertion within the lateral face area. None of the cadavers had any history of trauma, surgery, or deformity in the lateral facial region. This study received approval from the Institutional Review Board of the College of Medicine, The Catholic University of Korea (Approval No. MC22SISI0098). Written informed consent for the use of the cadavers and related materials in future research was obtained from all donors or their authorized representatives.

### Definition of the anatomical structures

**Figure 1:**
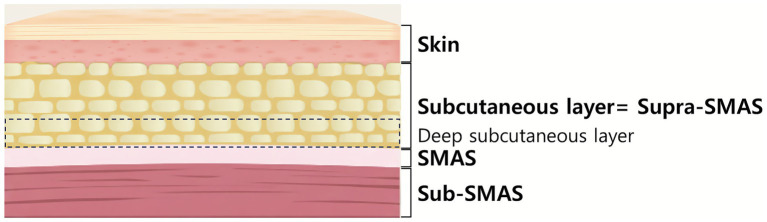
Schematic illustration depicting the basic anatomical layers of the face. The layers are presented from the skin to the sub-SMAS. The dotted square indicates the deep subcutaneous layer within the subcutaneous tissue, referred to as the supra-SMAS layer in this article. This deep subcutaneous layer is defined as the layer immediately superficial to the SMAS (superficial musculoaponeurotic system). Created using Gemini (https://gemini.google.com/).

Subcutaneous layer: between the dermis and SMAS (Figure 1).
Supra-SMAS: synonymous with the subcutaneous layer.
Deep subcutaneous: refers to the layer immediately above the SMAS, but deeper than the mid-dermal fat.
Near the SMAS: generalized term referring to the lower portion of the subcutaneous tissue close to the SMAS.

### Thread insertion and investigation via ultrasonography and micro-CT

**Figure 2:**
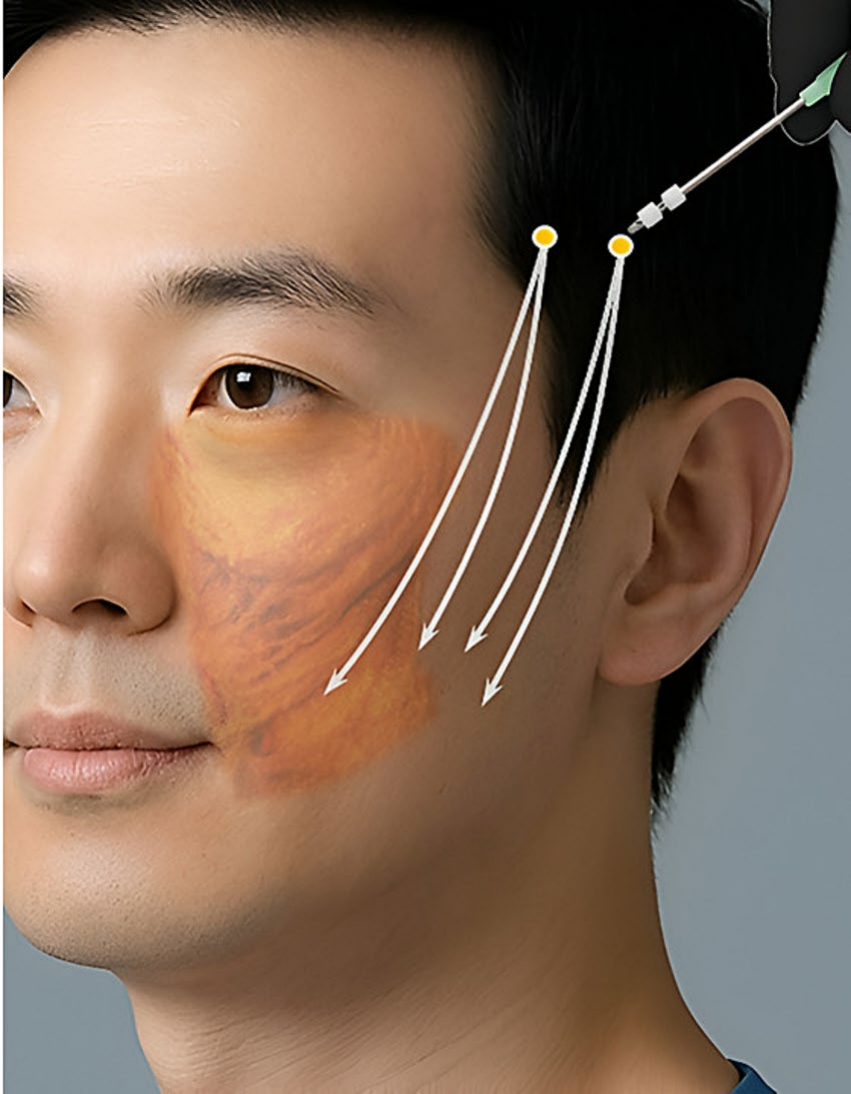
The thread pathways at the lateral face are used in the present study. Based on one entry point, three cannulas of 19-G with thread (N-Fix, N-Finders, Seoul, Korea) were inserted towards the mouth corner. Three cannulas were generally targeted to reach deep portion of the subcutaneous layer which might be considered the supraSMAS layer. Created using Gemini (Imagen 3) (https://gemini.google.com/).

The cadavers’ heads were positioned at a 45-degree downward angle to simulate the commonly used position in clinical settings. Threads were inserted following a typical thread-lifting path, as illustrated in Figure 1. One entry point was used: Two vertical reference lines were drawn, one passing through the lateral canthus and the other through the tragus. The area between these two lines was divided into five equal sections. A horizontal line passing through the eyebrow was drawn, and the intersection of this line with the fourth section served as the entry point. A 19-gauge cannula with thread (N-Fix, N-Finders, Seoul, Korea) was inserted from this point, and three threads were guided through the cannula to terminate at three target points: approximately 1 cm lateral to the corner of the mouth and two additional points, each 1 cm further laterally, targeting the lateral face area (Figure 2).

### Ultrasonography and micro-CT with a phosphotungstic acid agent

Ultrasonography was used to identify the location of the thread within the face. Real-time B-mode ultrasonography was performed with a linear array transducer (HS50, LA3-14 AD; 3–14 MHz, Samsung, Seoul, Republic of Korea). The threads were inserted without ultrasound guidance; subsequently, the insertion area was scanned to determine the precise anatomical layer of the face. To confirm the thread insertion layer, the ultrasonography transducer was positioned parallel to the cannula (in-plane method) containing the thread. After confirming the insertion layer, the cannula was removed, and the thread was pulled under ultrasonographic guidance to ensure advancement within the correct facial layer.

The cadavers’ heads were sectioned to facilitate phosphotungstic acid (PTA) staining and subsequent micro-CT scanning. Threads were inserted before hemi-sectioning the heads along the facial midline. Each hemi-section was further divided coronally along a vertical plane passing through the tragus. The procured specimens were fixed in 4% neutral buffered formalin for a week. Following fixation, tissues were dehydrated through a graded ethanol series (30 to 70%) over ten days. The samples were then immersed in a 1% PTA solution in 70% ethanol and gently agitated at 70 rpm on a shaker for 30 days. The PTA solution was refreshed every ten days to enhance penetration, and periodic gentle massages were applied to improve tissue absorption of the stain.

Data were obtained using a Nano & Microfocus X-ray CT system V|tome|x M (BakerHughes), which provides a minimum detectability of 0.2 μm, a voxel resolution of 0.5 μm, and an image resolution of 4,048 × 4,048 pixels at a maximum power of 300 kV/500 W. The three-dimensional (3D) reconstructed models were analyzed using 3D Slicer software (v.5.1.0, Slicer Community, www.slicer.org). These reconstructions were used to identify the location of the inserted thread within the lateral face area and their relationship between with surrounding anatomical structures, including subcutaneous tissue, facial muscles, and the SMAS.

Based on the micro-CT data, detailed anatomical structures were evaluated. In particular, the location of the facial nerve, parotid duct, and SMAS layer on the lateral face was investigated in detail.

For ultrasonography, each anatomical region was imaged three times. From each acquisition, three images were obtained, and the same anatomical structures were analyzed repeatedly by three anatomists on different days. For micro-CT, imaging data were acquired once per specimen, generating a complete 3D dataset. The three anatomists independently evaluated these datasets on multiple occasions, focusing on the same anatomical structures. To minimize recall bias, each evaluation was performed on different days. After independent assessments, all interpretations were compared, and the final analysis was based only on structures upon which all three anatomists reached consensus.

## Results

### The anatomical layers and contents of the lateral face

The lateral face consistently consisted of skin, subcutaneous, SMAS connected with platysma, and sub-SMAS layers containing the parotid-masseteric fascia, from superficial to deep in all cadavers (To simplify the description of the layered structures of the lateral face, we have omitted deeper anatomical structures beyond the sub-SMAS layer, including the parotid gland, facial nerve branches, masseter muscle, periosteum, mandible, etc.).

**Figure 3:**
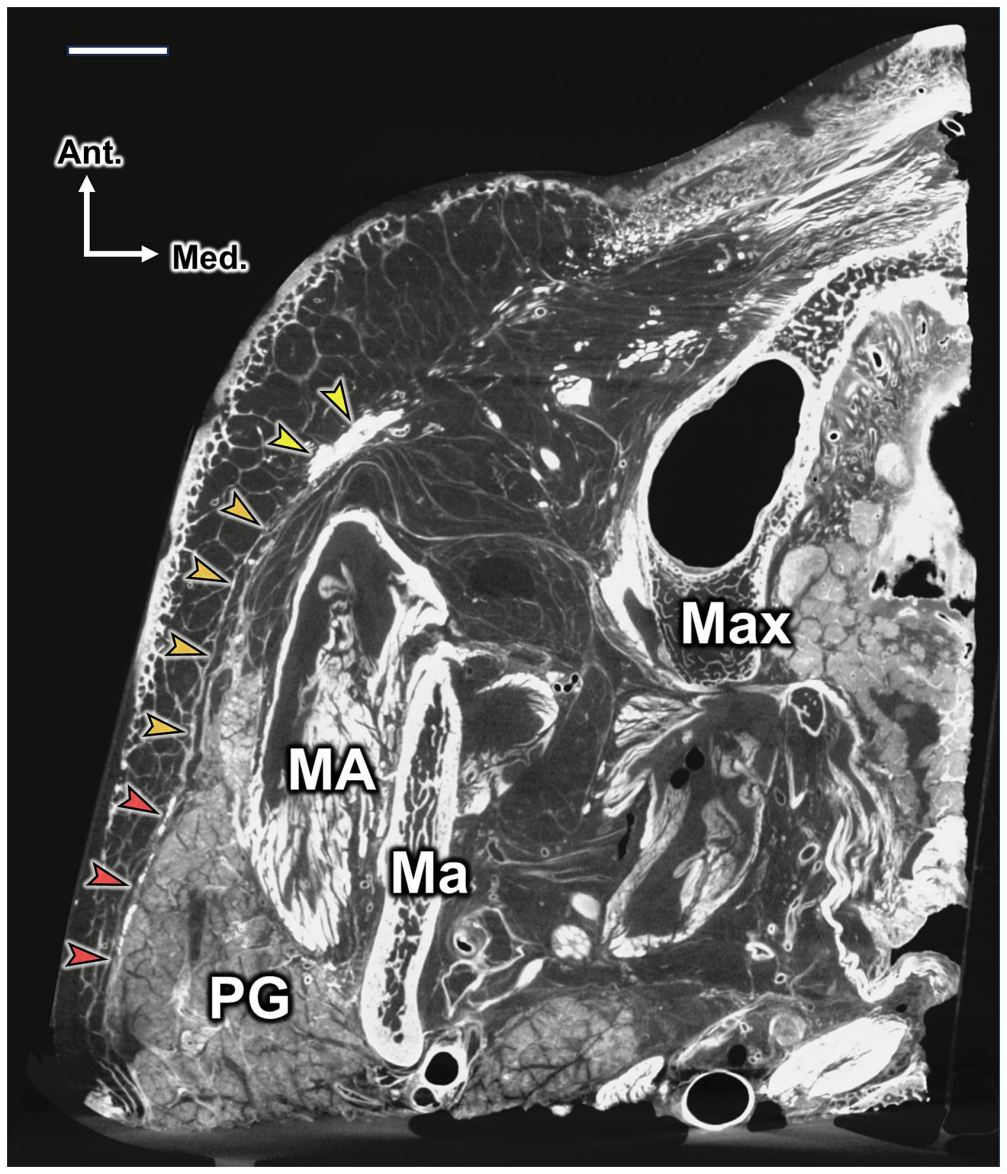
Micro-CT image showing the SMAS layer and its connections with the platysma and zygomaticus major and minor muscles. The image was taken at the level of the nasal ala. The red, orange, and yellow arrowheads indicate the platysma, SMAS, and zygomaticus major and minor muscles, respectively. Note that the platysma and SMAS cover the parotid gland (PG) and part of the masseter muscle (MA), with limited space between the SMAS, PG, and MA. Scale bar: 1 cm; Ma, mandible; Max, maxilla; Lat., lateral; Med., medial; Ant., anterior; Post., posterior.

Using micro-CT, the SMAS layer, along with the platysma and zygomaticus major and minor muscles, was clearly visualized from posterior to anterior (Figure 3). The facial nerve branches were located deep to the SMAS layer and superficial to the masseter muscle. Still, they became more superficial as they crossed the vertical line, passing through the lateral canthus. Due to the limited penetration of PTA into adipose-rich tissues, the distinct subcutaneous and SMAS layers within the lateral face could not be clearly visualized in the 3D reconstructed model. However, the lateral portions of the orbicularis oculi, zygomaticus major, and zygomaticus minor muscles were distinctly identified as whitish structures in the 3D model of the lateral face. The facial nerve branches ran deep into the zygomaticus major and minor muscle, and these branches were found deep into the SMAS layer.

**Figure 4:**
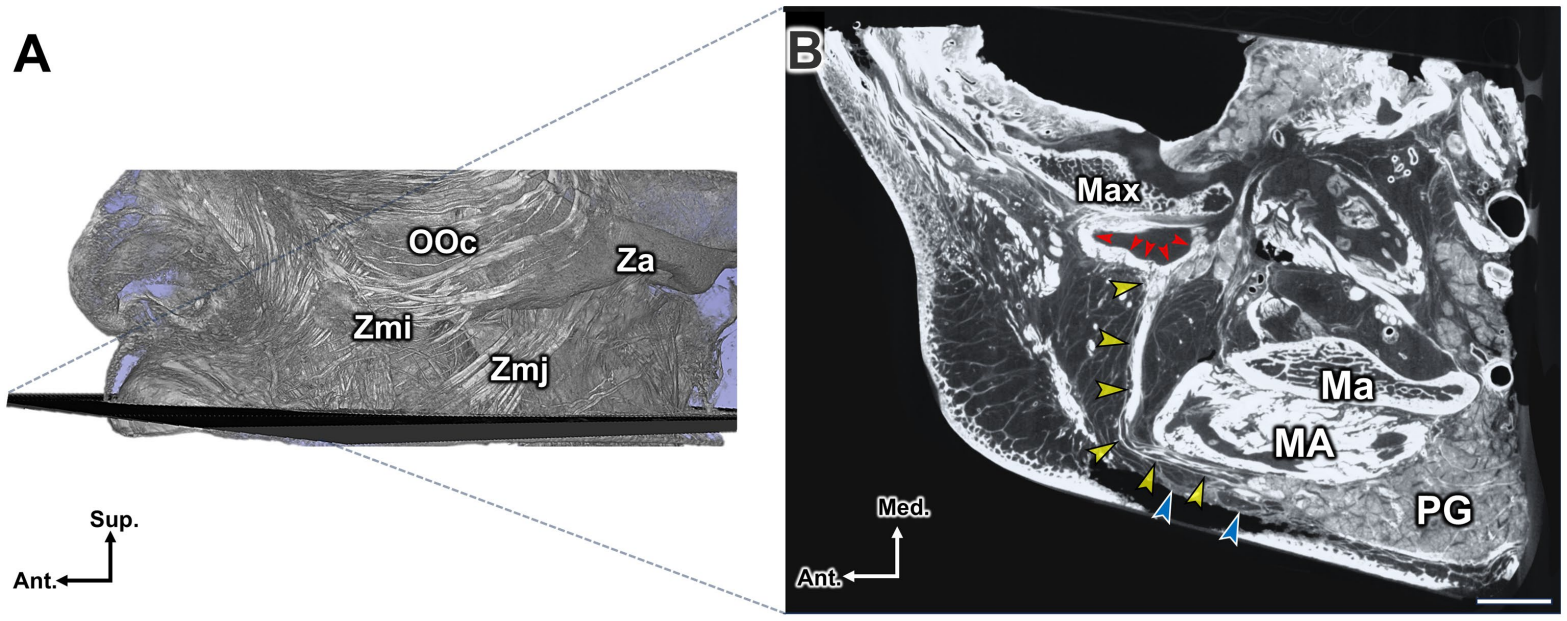
**(A)** The micro-CT image, captured at the level of a horizontal line passing through the otobasion inferius near the lower part of the ear (or the superior margin of the upper lip’s vermilion border), demonstrates the parotid duct coursing within the buccal fat pad and subSMAS layer. **(B)** The yellow arrowheads indicate the parotid duct, which emerges from the parotid gland (PG) located superficially and posterior to the masseter muscle (MA). The parotid duct travels anteriorly and medially within the buccal fat pad. After turning medially at the anterior portion of the masseter muscle, it penetrates the buccinator muscle (red arrowheads) to enter the oral cavity. The blue arrowheads denote the SMAS. Note the narrow space between the SMAS and the parotid duct. Scale bar: 1 cm; Ma, mandible; Max, maxilla. Lat., lateral; Med., medial; Ant., anterior; Post., posterior. Max, Maxilla; Ma, Mandible; MA, Masseter; PG, parotid gland.

The parotid duct was located deep into the SMAS and covered by the parotid-masseteric fascia. After emerging from the parotid gland, it ran superficial to the masseter muscle and curved medially to enter the oral cavity by penetrating the buccinator muscle (Figure 4).

### The location of the thread within the facial tissue using ultrasonography and micro-CT

**Figure 5:**
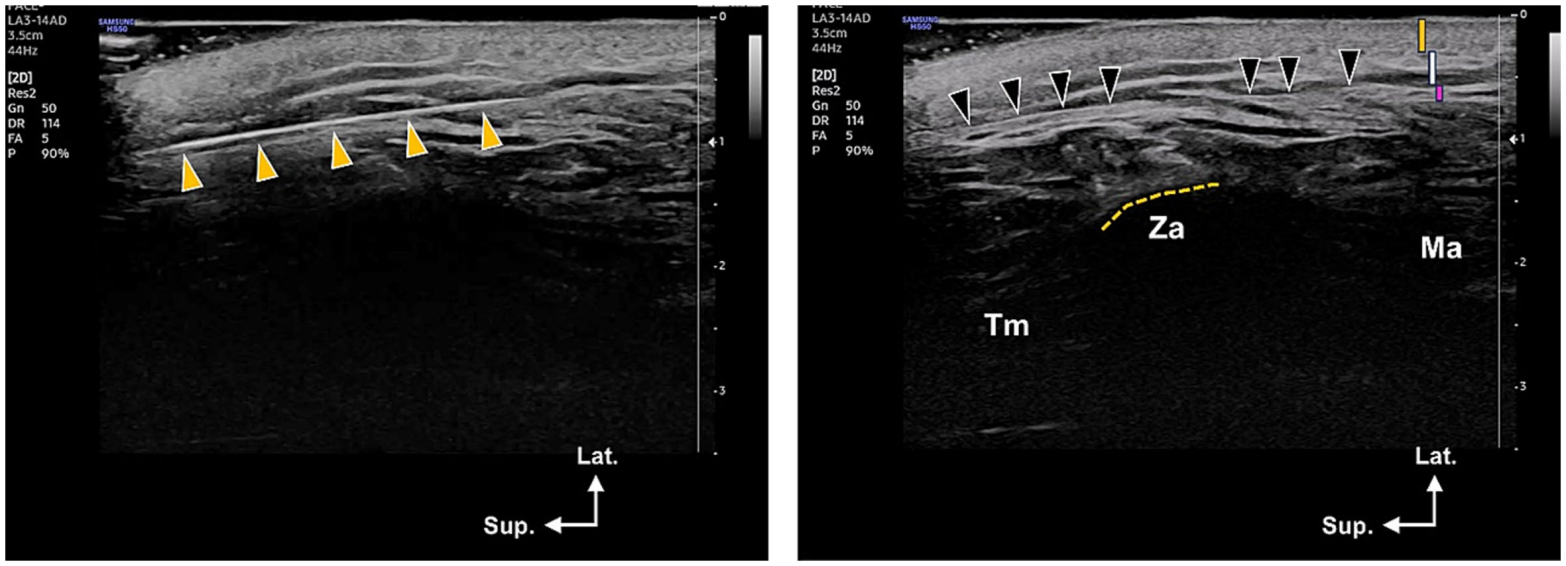
Ultrasonography images showing the location of the thread within the lateral face. The left image indicates the cannula’s location at the temple area, which was taken at the level of the lateral canthus. The yellow arrowheads point to the thread’s position within the temporoparietal or superficial temporal fascia. The right image displays the thread’s location within the subcutaneous layer (indicated by black arrowheads, which refer to the supra-SMAS in this article), specifically the deep portion of the subcutaneous layer just superficial to the SMAS layer. This image was taken at the zygomatic arch (ZA) level. In the upper right corner of the right image, the orange, white, and pink bars represent the skin, subcutaneous, and SMAS layers, respectively. Tm, Temporalis muscle; Ma, masseter muscle; Lat., lateral, Sup., superior.

Based on the anatomical layers, the threads were safely positioned within the Deep subcutaneous (used interchangeably with the term supra-SMAS in this article) of the lateral face. Ultrasonography and micro-CT imaging showed that the thread was located superficially near the entry point; however, as it advanced, the thread became deeper, ultimately reaching the deep subcutaneous layer at each termination point (Figure 5).

Measurements were obtained from contrast-enhanced micro-CT datasets using digital calipers in VGStudio MAX (Volume Graphics, Heidelberg, Germany). Distances were measured at two representative points along the mid-portion of the thread trajectory, and mean values were calculated. The mean thread insertion depth from the skin surface was 6.8 ± 1.1 mm, while the mean distance between the thread path and the SMAS plane was 1.2 ± 0.4 mm. However, minimal movement was observed in the deeper portions of the thread’s trajectory. Despite being inserted into the subcutaneous layer, there were no cases where the thread appeared through the skin, such as causing dimpling. After thread insertion and investigation within the lateral facial tissue, dissection of the specimens revealed no damage to vessels, nerves, and muscular structures in the lateral face area.

## Discussion

Our findings demonstrate that threads inserted into the subcutaneous layer of the lateral face can effectively avoid critical anatomical structures, such as the facial nerve, transverse facial artery, and parotid duct.

In facial thread lifting procedures, one of the most essential factors is the thread’s insertion layer within the face. Previous studies have reported that the Deep subcutaneous layer (used interchangeably with the term supra-SMAS in this article) is preferred because it helps avoid significant arteries and nerves in the lateral face, such as facial nerve branches and the superficial temporal and transverse facial arteries. However, in patients with less subcutaneous fat tissue (i.e., those with thinner facial structures), the supra-SMAS layer may be significantly superficial, approaching the skin. In such cases, threads inserted into the supra-SMAS layer close to the skin may become visible, thereby reducing the effectiveness of the facial thread lifting. In the present study, a tiny space existed between SMAS and the fascia covering the masseter muscle and parotid duct at the lateral face area, approximately near the zygomatic arch region. Therefore, it is recommended to insert the thread into an appropriate facial layer, which could be either the supra-SMAS layer or the deeper part of the subcutaneous layer (referring to the layer immediately above the SMAS, but deeper than the mid-dermal fat) that is very close to the SMAS. This approach allows clinicians to achieve a significant lifting effect while maintaining safety (9–11).

The facial nerve branches are among the most critical anatomical structures that must be meticulously considered during facial thread-lifting procedures. The facial nerve resides deep within the parotid gland before exiting it to traverse superficially over the masseter muscle. Subsequently, it pierces the fascia, covering the masseter muscle and parotid gland, distributing to most facial muscles. The transition point of the facial nerve—where it shifts from deep to superficial layers—has been identified (12). This transition point has been reported to occur approximately 2 centimeters anterior to the midpoint of the zygomatic arch, providing a crucial landmark for safe thread insertion. When inserting threads in the lateral face, it is crucial to avoid damaging the facial nerves near the zygomatic arch, specifically at the mid-face height of the lateral face. To minimize the risk of nerve injury, threads should not be inserted deeper than the zygomaticus major and minor muscles, nor deeper than the SMAS layer. Therefore, inserting threads at or above the SMAS layer is anatomically appropriate and safe, effectively reducing the risk of nerve damage while ensuring visible lifting effects. However, the choice of insertion layer—whether supra-SMAS, intra-SMAS, or sub-SMAS—should also consider the lifting effect and the degree of facial fat in patients. For instance, in patients with less subcutaneous fat, supra-SMAS insertion may be more effective without compromising safety, whereas in patients with more facial fat, deeper insertion near the SMAS may provide a more significant lifting effect. This flexibility allows clinicians to tailor their approach based on individual anatomical variations and esthetic goals, thereby enhancing the procedure’s efficacy and safety.

In addition to the insertion layer, the insertion method plays a critically important role in the success of facial thread lifting. The gentle insertion of the thread can help protect critical anatomical structures. Assisted by another operator’s hand, confirming or feeling the thread insertion layer during insertion could enhance the efficacy of the face thread lifting procedure and reduce the occurrence of serious iatrogenic complications. Given its sufficient tissue resistance and anchoring capacity, the subcutaneous layer supports prior research emphasizing it as a safe and mechanically effective plane for thread insertion. Unlike deeper layers within the SMAS or sub-SMAS, the subcutaneous layer offers minimal nerve, vessel, and parotid duct damage risk while enabling visible lifting effects. The consistency of these results across all cadaveric specimens highlights the robustness of this approach for thread-lifting procedures, even in the presence of anatomical variations.

Anatomical variability remains a critical consideration in thread lifting. Age-related changes in facial structures, such as thinning of the SMAS and redistribution of subcutaneous fat, can alter the mechanical properties of the targeted layer (13). These changes may affect thread anchoring and lifting outcomes, particularly in older individuals or patients with significant facial fat atrophy. Ethnicity is another key factor, as differences in skin thickness, SMAS characteristics, and fat layer composition have been shown to influence thread-lifting results (14). By standardizing thread insertion techniques using advanced imaging tools, such as ultrasonography or micro-CT, clinicians can better account for these variables, ultimately achieving safer and more predictable outcomes.

Micro-CT with PTA staining complemented the current study’s findings by offering high-resolution imaging for post-procedure assessment, revealing the spatial relationship between the thread and surrounding anatomical structures. Micro-CT has been widely used to demonstrate sophisticated anatomical structures of the head and neck area. Yi et al. revealed the minuscule larynx structure via micro-CT analysis with PTA (15). Furthermore, Kwon et al. investigated the nasolabial fold area using the micro-CT, and they unveiled the anatomical structure that contributes to the formation of the nasolabial fold with fat and muscular fiber attachment to the dermis (16). Despite its advantages, micro-CT’s inability to clearly differentiate subcutaneous and SMAS layers highlights the need for improved staining techniques or complementary imaging methods. Even though the agent used in the present study, the PTA, can significantly enhance soft tissue contrast compared to general CT, because of its characteristics, soft tissue cannot be distinguished from SMAS and fat tissue within the face.

One limitation of this study is the absence of formal inter- and intra-rater reliability analysis for the interpretation of micro-CT and ultrasonographic imaging. Although all images were independently evaluated multiple times by three experienced anatomists, and the final interpretations were based only on structures unanimously agreed upon through consensus, quantitative measures of agreement (e.g., intraclass correlation coefficients or kappa statistics) were not calculated. This may limit the assessment of reproducibility and objectivity in imaging interpretation. Future studies should incorporate standardized reliability assessments to validate measurement consistency across observers. Another limitation of this study is that all cadaveric specimens were elderly (mean age: 72.1 years), which may not accurately reflect the anatomical characteristics of younger individuals who typically undergo thread lifting. Age-related changes—such as thinning of the SMAS, attenuation of retaining ligaments, and superficial fat compartment contraction—may reduce mechanical resistance and increase the risk of contour irregularities following thread placement. Additionally, all specimens were of Korean origin, which may limit the generalizability of our findings to populations with different craniofacial architectures, SMAS morphology, and fat distribution patterns. Ethnic variability in facial soft tissue layers and ligamentous support should be accounted for in future anatomical and clinical validations to optimize thread lifting strategies across diverse patient groups.

While this study provides a robust anatomical basis for thread lifting in the lateral face, it is limited to using cadaveric models, which may not fully replicate *in vivo* conditions. Future research should include clinical trials to evaluate the long-term outcomes of thread placement in different anatomic layers and thread products in living subjects. Expanding the sample size to include more diverse demographic groups would provide a broader understanding of anatomical variability. Further investigations into the effects of thread placement on lifting efficacy and patient satisfaction will be crucial for advancing the field.

## Conclusion

This study highlights the critical anatomical considerations for lateral face thread lifting, demonstrating that the subcutaneous layer can be one of the relatively safe planes for thread placement without evident arterial or nerve injury. Nonetheless, the proximity of superficial temporal artery branches, facial nerve branches in the temple area, and deeper structures such as the parotid gland and masseter muscle necessitates a thorough understanding of facial anatomy. Therefore, gentle thread insertion would allow clinicians to prevent dimpling and reduce the risk of inadvertent tissue damage, thereby enhancing the lifting efficacy. These findings provide a robust anatomical foundation for improving clinical practices and advancing the field of minimally invasive facial rejuvenation.

## Data Availability

The data that support the findings of this study are available from the corresponding author upon reasonable request.
